# Tetralogy of Fallot: Genetic, Epigenetic and Clinical Insights into a Multifactorial Congenital Heart Disease

**DOI:** 10.3390/genes17020181

**Published:** 2026-01-31

**Authors:** Maria Felicia Gagliardi, Emanuele Micaglio, Angelo Micheletti, Sara Benedetti, Diana Gabriela Negura, Francesca Bevilacqua, Giulia Guglielmi, Giulia Pasqualin, Alessandro Giamberti, Massimo Chessa

**Affiliations:** 1 Faculty of Medicine and Surgery, Milano-Bicocca University, 20126 Milan, Italy; 2 Pediatric and Adult Congenital Heart Centre, IRCCS Policlinico San Donato, 20097 Milan, Italy; 3 Arrhythmia and Electrophysiology Department, IRCCS Policlinico San Donato, 20097 Milan, Italy; 4 Postgraduate School in Radiodiagnostics, University of Milan, 20132 Milan, Italy; 5 Congenital Cardiac Surgery Unit, IRCCS Policlinico San Donato, 20097 Milan, Italy; 6 Faculty of Medicine and Surgery, Vita-Salute San Raffaele University, 20132 Milan, Italy

**Keywords:** tetralogy of Fallot, congenital heart disease, genetics, NOTCH1, FLT4, epigenetics, genotype–phenotype correlation, precision medicine

## Abstract

Tetralogy of Fallot (TOF) is the most common cyanotic congenital heart disease, classically characterized by right ventricular outflow tract obstruction, ventricular septal defect, overriding aorta, and right ventricular hypertrophy. Recent advances in molecular and genomic research indicate that TOF is part of a phenotypic continuum encompassing Trilogy, Tetralogy, and Pentalogy of Fallot, in which the variability of anatomical presentation reflects shared genetic and epigenetic mechanisms with highly variable penetrance and expressivity. Variants in *NOTCH1*, *FLT4*, *KDR*, *GATA6*, and *TBX1* highlight key pathways in conotruncal development and endothelial–mesenchymal transition, yet these well-known genes explain only a fraction of the genetic landscape. Emerging studies have identified additional candidate genes and networks involved in cardiac morphogenesis, including transcriptional regulators, signaling mediators, chromatin-remodeling factors, and splicing-associated genes such as PUF60 and DVL3. Epigenetic mechanisms, including DNA methylation, histone modifications, and non-coding RNA expression, further modulate phenotypic expressivity and contribute to variability along the Trilogy–Tetralogy–Pentalogy spectrum. This review integrates current genomic and clinical evidence to provide a comprehensive overview of the molecular architecture of Fallot-type conotruncal malformations, emphasizing the interplay between genetic and epigenetic mechanisms, genotype–phenotype correlations, and implications for diagnosis, risk stratification, counseling, and personalized management in the era of precision cardiology.

## 1. Introduction

Tetralogy of Fallot (TOF) is the most common cyanotic congenital heart disease, accounting for approximately 7–10% of all congenital heart defects (CHDs) [1]. The estimated birth prevalence of TOF ranges from about 3 to 6 per 10,000 live births worldwide [2]. Advances in prenatal diagnosis and surgical repair have significantly improved survival, with long-term survival rates reported around 85% in earlier cohorts [2]. TOF occurs slightly more frequently in males than females, although many studies report near-equal sex distribution. While most cases are sporadic, familial recurrence has been reported in approximately 3% of affected families, indicating a contribution from genetic factors [1,2].

**Figure 1:**
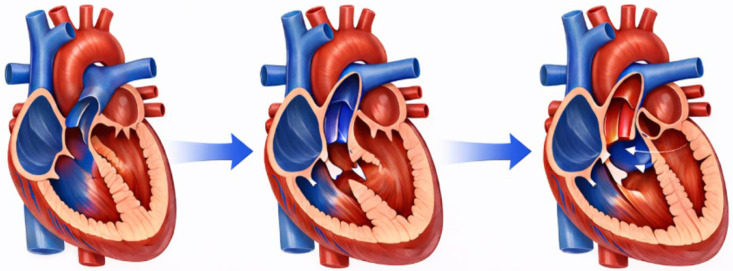
Phenotypic severity progresses from Trilogy to Tetralogy to Pentalogy of Fallot in association with an increasing cumulative genetic and epigenetic burden. The blue arrow represents the spectrum of Fallot from the less to the more severe phenotype (i.e., from Trilogy to Pentalogy).

From a structural perspective, TOF is a conotruncal cardiac defect defined by a large, anteriorly misaligned ventricular septal defect, an overriding aortic root, and varying degrees of narrowing of the sub pulmonary region and pulmonary valve, with secondary right ventricular hypertrophy resulting from obstruction of the right ventricular outflow tract (RVOT). The severity and clinical presentation depend primarily on the extent of RVOT obstruction, the balance of intraventricular pressures, and the degree of aortic override [1,3,4,5,6]. Increasing evidence indicates that Fallot-type congenital heart disease arises along a developmental and genetic continuum driven by progressively more disruptive genomic alterations, ranging from modest copy-number variants and low-impact multigenic hits to high-penetrance structural variants or pathogenic mutations in key conotruncal pathways [7,8,9,10,11]. Within this spectrum, the trilogy of Fallot—typically consisting of pulmonary stenosis, right ventricular hypertrophy, and an atrial septal defect—appears to arise from comparatively milder genomic perturbations that modestly influence outflow tract development. As the cumulative genetic and epigenetic burden increases, involving more consequential CNVs or deleterious variants in genes that regulate key conotruncal developmental pathways (including vascular signaling, cell-fate specification, and ciliary function [3,4,5,6,11], the phenotype progresses toward the classical tetralogy of Fallot, in which the full set of structural defects—ventricular septal defect, overriding aorta, pulmonary stenosis, and right ventricular hypertrophy—is fully expressed. When an additional septal defect or other outflow tract anomaly is present, the spectrum culminates in the pentalogy of Fallot, representing the most complex form of this genetically mediated conotruncal abnormal development (Figure 1). We emphasize that this continuum does not imply a deterministic relationship between specific genetic variants and distinct anatomical subtypes. Rather, it reflects a multifactorial model in which overlapping mechanisms—including copy-number variations, point mutations, mosaicism, and epigenetic modifications—collectively shape phenotypic expression. In practice, the same genetic alteration may result in different manifestations across individuals, and no studies have identified unique genetic signatures specific to Trilogy of Fallot.

Overall, Trilogy, Tetralogy, and Pentalogy should be viewed as gradations within a shared pathogenic continuum, in which the cumulative effect of structural variants, rare coding mutations, and multigenic susceptibility modulates phenotype, while allowing for considerable variability in clinical presentation [7,8,9,10,11].

## 2. Genetic Architecture and Pathophysiology of Tetralogy of Fallot

Although TOF has traditionally been considered a sporadic congenital malformation, accumulating evidence from high-throughput sequencing and genome-wide studies indicates that it has a significant genetic basis, encompassing both monogenic variants and a multifactorial polygenic background [3].

### 2.1. Mutations and Associated Genes

The genetic basis of TOF, which arises during the early stages of embryonic development, is not yet fully understood. Nevertheless, pathogenic variants have been identified in numerous genes involved in cardiac morphogenesis, transcriptional regulation, and signal transduction. Most of this evidence comes from studies in human patients. The most frequently implicated genes include:

- **Transcription factors:** *NKX2-5*, *GATA4*, *GATA6*, *TBX1*, *TBX5*, and *ZFPM2* (*FOG2*), often affecting cardiac progenitor differentiation [3,4,5,6].
- **Signaling pathway components:** *NOTCH1*, *JAG1*, *FLT4* (*VEGFR3*), *NFATC1*, *PTPN11*, *PDGFRA*, and *SMAD2/4*, disrupting endothelial–mesenchymal transition and outflow tract septation [3,4,5,6].
- **Structural and developmental genes:** *MEIS2*, *SOX11*, *FLRT2*, *FKBP10*, *MST1R*, and *GNE*, identified in recent sequencing studies and thought to influence both cardiac and neural crest development [12,13,14].

Moreover, recent exome-sequencing studies in patients with non-isolated Tetralogy of Fallot (TOF+) have identified PUF60 and DVL3 as emerging candidate genes [12]. PUF60 (Poly-U Binding Splicing Factor 60 kDa) encodes a protein that is part of the spliceosome, a cellular complex essential for pre-mRNA splicing. Proper splicing is critical during embryogenesis, including the development of the heart. In TOF+, PUF60 variants may disrupt RNA processing in cardiac progenitor cells, leading to abnormal cardiac morphogenesis. Heterozygous mutations or deletions in PUF60 cause Verheij syndrome, characterized by psychomotor delay, short stature, and septal heart defects. The observation of congenital heart defects in these patients suggests that PUF60 may contribute to conotruncal malformations, including TOF+. However, at present, functional studies in human cardiomyocytes or animal models are lacking, and the association is largely based on bioinformatic analyses and genotype–phenotype correlation. PUF60 was prioritized for study due to its role in RNA splicing, its involvement in a syndrome that includes cardiac defects, and its developmental relevance to conotruncal morphogenesis [12,15].

DVL3 (Disheveled Segment Polarity Protein 3) encodes a key mediator of the Wnt signaling pathway, which is essential for regulating cardiac progenitor cell proliferation, migration, and differentiation during the development of the outflow tract. Heterozygous mutations or deletions in DVL3 cause Robinow syndrome, often associated with macrocephaly, genital anomalies, and congenital heart defects. Given the frequent occurrence of conotruncal defects in these patients, DVL3 has been proposed as a candidate gene for non-isolated TOF [12,16,17]. As with PUF60, direct experimental validation in cardiac models is not yet available, and current evidence is mainly derived from bioinformatic analyses and the observed phenotypic overlap.

In addition to single-gene variants, copy-number variants (CNVs) play a significant role in TOF pathogenesis. The most clinically relevant is the 22q11.2 deletion, where haploinsufficiency of *TBX1* is a major driver of conotruncal defects. Studies in human patient cohorts have shown that additional dosage-sensitive genes within the critical region—including *DGCR8*, *CRKL*, and *MAPK1*—also contribute substantially to the TOF phenotype through combined disruption of microRNA biogenesis, FGF–MAPK signaling, and neural crest development [7,8,10].

Furthermore, large-cohort studies have shown that ciliary dysfunction substantially increases the risk of TOF. This effect is linked to loss-of-function variants in genes related to both primary and motile cilia—such as *DNAH11*, *DYNC2H1*, *C2CD3*, *OFD1*, and other intraflagellar transport (IFT) components—which can disrupt left–right body patterning, flow-dependent signaling, and Hedgehog/Notch pathways, all of which are crucial for proper development of the cardiac outflow tract [11].

Mutations may be loss-of-function (nonsense, frameshift, splicing) or missense variants altering protein–protein interactions in developmental signaling networks. Published human exome sequencing data indicate that rare deleterious variants in specific genes account for a measurable proportion of isolated TOF. In the largest cohort reported to date (n = 829), unique deleterious variants in NOTCH1 were found in ~4.5% of individuals and in FLT4 in ~2.4%, with these two loci together explaining ~7% of non-syndromic TOF cases. When expanding to the top nine candidate genes, approximately 15–16% of non-syndromic TOF patients carried deleterious variants in at least one candidate gene, supporting a substantive, though incomplete, monogenic contribution. The remaining cases likely reflect polygenic inheritance and epigenetic mechanisms, highlighting the multifactorial nature of isolated TOF [4].

Sex-related differences in TOF prevalence have been observed, with males slightly more affected than females. Genetic evidence suggests that X-linked mechanisms may contribute to this bias. A whole-exome sequencing study in an Iranian family with non-syndromic TOF identified a novel hemizygous mutation in *FLNA*, an X-linked gene essential for outflow tract development, supporting the role of X-linked variants in the increased susceptibility of males. Because males have a single X chromosome, deleterious *FLNA* variants are more likely to produce clinical effects [18].

### 2.2. Inheritance Patterns

Most TOF cases occur sporadically, resulting from de novo or incompletely penetrant autosomal dominant in genes such as *NKX2-5*, *GATA4*, and *NOTCH1* [3,4]. Autosomal dominant transmission is occasionally observed in familial cases, reflecting variable expressivity and incomplete penetrance [3,4]. Chromosomal abnormalities such as 22q11.2 deletion syndrome (DiGeorge/velocardiofacial) account for 10–15% of cases and follow autosomal dominant inheritance with variable expressivity [3,4]. A minority of forms display autosomal recessive inheritance, notably those involving *MST1R* and *FKBP10* [13,14]. Finally, polygenic inheritance is likely responsible for most isolated forms, reflecting the cumulative effect of rare deleterious variants interacting with common susceptibility alleles [6].

### 2.3. Pathophysiological Pathways

**Figure 2:**
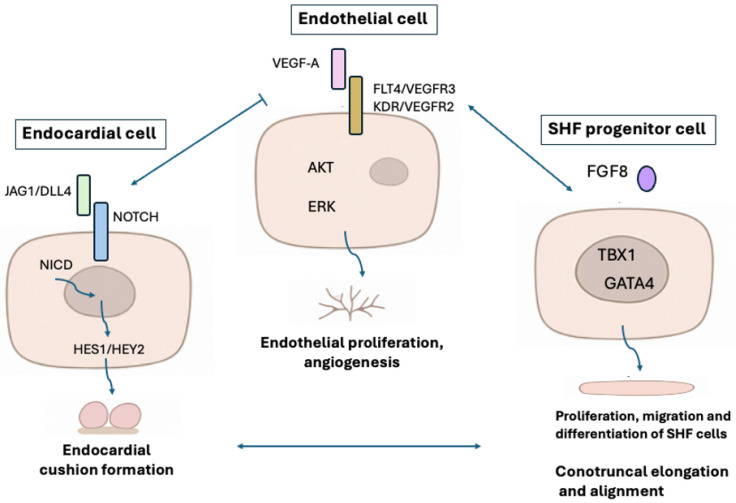
Crosstalk among Notch, VEGF/FLT4-KDR and SHF pathways regulating conotruncal elongation and alignment. Dysregulation leads to Tetralogy of Fallot. The light blue arrows represent the different mechanisms by which the Tetralogy phenotype might result.

The principal molecular pathways implicated in TOF pathogenesis include the Notch signaling pathway (*NOTCH1*, *JAG1*), essential for endocardial cushion formation and outflow tract septation [4,6]; the VEGF/FLT4 pathway, regulating endothelial proliferation and angiogenic remodeling [5]; and the second heart field (SHF) network (*TBX1*, *FGF8*, *GATA4*), crucial for conotruncal elongation and alignment [3,4] (Figure 2). Genes such as *MEIS2*, *SOX11*, and *FLRT2* influence neural crest migration and its interaction with the SHF [6,14]. Additionally, dysregulation of calcium–NFAT and MAPK pathways has been observed in transcriptomic analyses of TOF myocardium, linking developmental defects to maladaptive postnatal remodeling [6,8].

Collectively, these findings highlight that TOF results from the convergence of multiple disrupted developmental pathways—Notch, VEGF, and SHF–neural crest signaling—rather than single-gene defects, emphasizing the need for integrative genomic and transcriptomic approaches to fully elucidate its multifactorial etiology [3,4,5,6]. Together, these pathways converge on disrupted interaction between the second heart field and cardiac neural crest, leading to malalignment of the outflow tract and the classic anatomic features of TOF: ventricular septal defect, overriding aorta, right ventricular outflow obstruction, and hypertrophy [1].

### 2.4. Neuro–Cardiac Interactions and Emerging Genetic Determinants of Neurological Risk in TOF

Recent evidence indicates that neurological vulnerability in patients with Tetralogy of Fallot (TOF) may not be solely determined by perioperative or hemodynamic factors but could also involve specific genetic susceptibilities. *GPR91* (SUCNR1), a succinate receptor responsive to metabolic stress and hypoxia, has been identified through bioinformatic analysis as a potential key gene mediating brain injury under low-flow, hypothermic conditions [19]. At present, there is no evidence suggesting a direct role in cardiac morphogenesis or conotruncal malformations, including TOF. Its potential clinical relevance relates to neurological complications associated with chronic cyanosis in TOF patients: variants in GPR91 may modulate neuronal responses to hypoxic stress, influencing individual susceptibility to cerebral injury. This hypothesis is also based on bioinformatic prediction rather than experimental validation.

Furthermore, several genes implicated in TOF pathogenesis, including *NOTCH1*, *FLT4*, *MEIS2*, and *SOX11*, also play critical roles in neural crest development and blood–brain barrier integrity. This convergence suggests that a subset of TOF patients may possess shared neurogenetic vulnerabilities, in which disruptions of cardiovascular development and neurovascular protection co-occur [3,4,6,7,8,9,12].

Understanding these genetic interactions provides a framework for integrating cardiovascular and neurodevelopmental genetics, potentially informing neurological risk stratification and neuroprotective strategies in patients with TOF.

## 3. Epigenetic Regulation in Tetralogy of Fallot

Epigenetic mechanisms have emerged as crucial modulators of gene expression during cardiac morphogenesis and are increasingly recognized as contributors to the pathogenesis of Tetralogy of Fallot (TOF) [20,21]. These mechanisms—principally DNA methylation, histone modification, and non-coding RNA regulation—modulate transcriptional activity without altering the DNA sequence, thereby influencing key developmental pathways such as *NOTCH*, *TBX1*, and *GATA* signaling, which are essential for outflow tract septation and proper ventricular alignment [3,4,20].

Several lines of evidence indicate that genetic mutations explain only a small proportion of CHD cases, whereas aberrant expression regulated by epigenetic modifications plays a predominant role. For example, signaling pathways including Sonic hedgehog, retinoic acid, Wnt family members, Nodal growth differentiation factors, and bone morphogenetic proteins regulate transcription factors in the posterior mesoderm such as *NKX2-5*, *GATA4*, *ISL1*, and *TBX1*. Disruption of this complex machinery alters gene expression without changing the DNA sequence, suggesting that epigenetic alterations can precede structural malformations [20]. Even in DiGeorge patients, the deleted region may include genes involved in histone modification, such as *CDC45* and *TUPLE1*, highlighting the clinically pivotal role of epigenetic signatures in CHD [22]. Recent evidence further indicates that epigenetic alterations in TOF are cell-type-specific, affecting epithelial, neural crest, and endothelial lineages in distinct ways, consistent with the multifaceted developmental origin of the outflow tract [23,24]. It is important to note that many epigenetic studies in TOF have analyzed myocardial tissue obtained at surgical repair or postnatal blood samples [20,23,24]. While informative, these tissues may reflect secondary changes from chronic hypoxia or altered hemodynamics rather than primary developmental defects. Only a few studies have assessed fetal or early postnatal tissues, which are more informative regarding the timing of epigenetic dysregulation relative to cardiac morphogenesis [23,24].

### 3.1. DNA Methylation

Genome-wide methylation studies in TOF myocardium and blood samples have revealed distinct methylation signatures compared with controls [20,21]. Hypermethylation of promoter regions in cardiac transcription factors including *NKX2-5*, *HAND1*, and *GATA4* has been shown to repress their expression, leading to impaired cardiomyocyte differentiation and abnormal conotruncal morphogenesis [3,20]. In contrast, hypomethylation of genes involved in angiogenesis, such as *VEGFA* and *FLT4*, suggests compensatory activation of vascular remodeling pathways [5,6]. Studies of fetal heart tissue indicate that altered methylation within the NOTCH and VEGF loci occurs during early cardiac development, supporting a role for epigenetic dysregulation that may precede structural malformations rather than arising solely because of postnatal hemodynamic stress [6,21].

Recent integrative analyses combining transcriptomic, DNA methylation, and single-cell datasets in Tetralogy of Fallot (TOF) patients identified four candidate genes (*GJA1*, *SFRP1*, *PRICKLE1*, *PTK7*) showing promoter hypermethylation and altered expression in second heart field progenitors and neural crest cells. This evidence suggests that transcriptional repression of these polarity regulators may contribute to TOF pathogenesis [23].

A complementary study of newborn blood methylomes revealed endothelial-specific methylation differences affecting genes in VEGF, NOTCH, and PI3K–Akt pathways, further supporting an early developmental origin of epigenetic deregulation affecting endocardial and vascular cell populations [24].

Although functional data remain limited, a recent editorial has drawn attention to *MYH6* variants as possible contributors to Tetralogy of Fallot, suggesting that alterations in sarcomeric gene expression may play a role in cardiac malformations [25].

### 3.2. Histone Modifications

Chromatin remodeling also plays an important role in Tetralogy of Fallot (TOF). Studies have shown that the *TBX1* gene can promote histone deacetylation at the *MEF2C* enhancer, causing a reduction in H3 acetylation and silencing *MEF2C* expression. Since *MEF2C* is essential for the proper development of the second heart field (SHF), this mechanism contributes to the cardiac malformations observed in TOF. These data come primarily from animal models, and their direct relevance to early human cardiac development is inferred rather than directly observed [26]. Other reviews on the epigenetic regulation of TOF report that a loss of activating histone marks (such as H3K27ac) and an increase in repressive marks (such as H3K9me3) can suppress key cardiac transcription factors like *TBX1* and *GATA4* [3,20]. It remains unclear whether these histone changes precede malformations in humans or reflect postnatal adaptation. Altogether, these findings indicate that alterations in histone-modifying enzymes, including histone deacetylases (HDACs) and methyltransferases (KMTs), may contribute to conotruncal defects and could represent potential targets for future epigenetic therapies [3,20,26].

### 3.3. Non-Coding RNAs

MicroRNAs (miRNAs) and long non-coding RNAs (lncRNAs) have been identified as additional regulators of cardiac gene expression in TOF [3,20]. Downregulation of *miR-1* and *miR-133* disrupts normal expression of *HAND2* and *GJA1*, leading to abnormal myocardial proliferation and septation defects. These findings are primarily derived from postnatal cardiac tissues, and it is not yet established whether these miRNA changes occur before or after the development of structural defects [3,20]. Moreover, dysregulated *miR-424* and *miR-222* modulate endothelial and smooth muscle cell differentiation within the outflow tract [20]. Long non-coding RNAs such as *HOTAIR* and *MALAT1* interact with chromatin-remodeling complexes and modulate *NOTCH1* and *GATA4* expression, further influencing conotruncal development [3,20].

### 3.4. Cell-Type-Specific Epigenetic Alterations: Epithelial vs. Endothelial Contributions

Emerging multi-omics studies highlight that epigenetic dysregulation in TOF is not uniform across cardiac tissues but affects distinct developmental lineages:

- **Epithelial and Neural Crest Epigenetic Alterations:** Bioinformatic and integrative analyses of TOF heart tissues identified hypermethylation and expression changes in epithelial- and neural crest-related genes, including GJA1, SFRP1, PRICKLE1, and PTK7. These genes regulate epithelial polarity, PCP signaling, SHF patterning, and neural crest migration, suggesting that impaired epithelial organization and SHF–neural crest interaction contribute to conotruncal misalignment [23].
- **Endothelial Epigenetic Alterations:** A methylome-wide study of newborn blood identified significant differential methylation in endothelial genes involved in VEGF, NOTCH, and PI3K–Akt signaling. These pathways regulate endocardial cushion formation, angiogenesis, and outflow tract vascularization, indicating that endothelial epigenetic perturbations are an early and distinct contributor to TOF [24].

### 3.5. Rare Association of the Fallot Spectrum with Systemic Epigenetic Disorders

Although tissue-specific and developmental epigenetic dysregulation is increasingly recognized in TOF, Tetralogy of Fallot is extremely rare in classical systemic epigenetic disorders (e.g., imprinting diseases). To date, the literature documents only a single well-characterized case, reported in 2009, describing TOF in a patient with Beckwith–Wiedemann syndrome, an imprinting disorder involving dysregulation of chromosome 11p15.5 (including H19/IGF2) [27].

This observation supports the idea that global imprinting defects do not constitute a common etiological mechanism for the Fallot spectrum. Instead, TOF-related epigenetic changes appear to arise from localized, lineage-specific alterations affecting epithelial, endothelial, and neural crest developmental programs rather than from systemic epigenetic disorders.

## 4. Mitochondrial Dysfunction and Redox Imbalance in Tetralogy of Fallot

Growing evidence indicates that mitochondrial dysfunction contributes to the pathophysiology of Tetralogy of Fallot (TOF), particularly because it is a cyanotic congenital heart disease characterized by chronic hypoxia and altered redox homeostasis. Mitochondrial DNA (mtDNA) is highly vulnerable to oxidative stress due to its close proximity to the electron transport chain and limited repair capacity, leading to oxidative damage, copy-number instability, and impaired expression of respiratory chain components. Consistently, right ventricular tissue from TOF patients shows significant reductions in the activity of multiple mitochondrial respiratory chain enzymes—especially complexes I and IV—suggesting compromised oxidative phosphorylation and bioenergetic stress [28,29]. These alterations are further supported by broader studies in cyanotic congenital heart disease, which demonstrate hypoxia-driven defects in mitochondrial dynamics, ATP production, and metabolic reprogramming [29]. These findings primarily reflect postnatal and chronic adaptive responses to sustained hypoxemia. Accordingly, broader studies in cyanotic congenital heart disease demonstrate hypoxia-driven defects in mitochondrial dynamics, ATP production, and metabolic reprogramming that emerge after birth and progress over time [29]. In this postnatal context, experimental studies using cardiac-specific murine models have identified Forkhead box protein O1 (FOXO1) as a key mediator of hypoxia-induced mitochondrial dysfunction in cardiomyocytes. FOXO1 acts as a metabolic stress sensor, orchestrating transcriptional responses that modulate mitochondrial homeostasis and cell survival under chronic hypoxic conditions, thereby contributing to postnatal myocardial vulnerability in TOF [30]. In contrast, mitochondrial dysfunction in TOF is not exclusively a secondary or ac postnatal adaptive phenomenon.

Recent developmental studies have shown that NOTCH1 can localize to mitochondria in embryonic endocardial cells, where it promotes mitochondrial oxidative metabolism and influences endothelial-to-mesenchymal transition, a key process in outflow tract and valve development [31].

This observation links mitochondrial metabolism not only to postnatal cyanosis-related changes but also to embryonic morphogenetic pathways directly relevant to TOF.

## 5. Genotype–Phenotype Correlations

### 5.1. Genotype–Phenotype Correlations in Tetralogy of Fallot

The genotype–phenotype correlation in Tetralogy of Fallot is not univocal, as the heterogeneous clinical presentations reflect a complex multifactorial interplay among genetic, epigenetic, and environmental factors influencing cardiac development. Even within families sharing the same mutation, variable expressivity and incomplete penetrance are common, reflecting the influence of epigenetic modifiers and gene–environment interactions [20]. For example, differential methylation of *NKX2-5* and *TBX1* loci or altered expression of microRNAs such as *miR-1* and *miR-133* can modulate the degree of right ventricular hypertrophy and cyanosis [21,27].

From a molecular standpoint, at least four major mechanisms contribute to TOF pathogenesis: copy number variations, point mutations, mosaicism, and epigenetic alterations. Importantly, these mechanisms appear to operate in a largely overlapping manner in both isolated and syndromic TOF, rather than defining discrete anatomical subtypes. For instance, loss-of-function variants of TBX1 have been identified in a subset of patients with isolated TOF [32]. While TBX1 haploinsufficiency is a well-established pathogenic mechanism in murine models [33], in humans it exhibits considerable phenotypic variability and limited predictive value for specific anatomical configurations.

Despite this complexity, certain genotypes have been associated with particular anatomical or functional features, indicating that some molecular variants can exert targeted effects on cardiac morphogenesis. However, these associations should not be interpreted as deterministic or as defining discrete TOF subtypes. Current evidence does not support the existence of genetic signatures that reliably distinguish Trilogy, Tetralogy, or Pentalogy of Fallot as separate molecular entities. Rather, these anatomical variants appear to reflect overlapping manifestations of shared developmental perturbations, characterized by variable penetrance and expressivity.

### 5.2. Syndromic Versus Non-Syndromic Forms

Tetralogy of Fallot (TOF) can manifest as either a non-syndromic isolated cardiac malformation or as part of a syndromic condition involving multiple organ systems. Approximately 15–25% of TOF cases occur in the context of a genetic syndrome, where chromosomal deletions or monogenic mutations disrupt developmental programs that extend beyond the heart, resulting in systemic phenotypic manifestations alongside congenital cardiac malformations [34,35,36].

The 22q11.2 deletion is the most common syndromic association, present in up to 15% of TOF cases [37]. This microdeletion affects multiple genes crucial for pharyngeal arch and outflow tract development, most notably TBX1, a key regulator of second heart field (SHF) proliferation and conotruncal septation [27]. Additionally, other dosage-sensitive genes within the critical region—*DGCR8*, *CRKL*, and *MAPK1*—contribute significantly to the TOF phenotype through effects on microRNA biogenesis, FGF–MAPK signaling, and neural crest development, modulating the severity and complexity of cardiac malformations [10]. Clinically, individuals with 22q11.2 deletions often present with hypocalcemia, thymic hypoplasia, velopharyngeal insufficiency, and characteristic facial dysmorphisms, with more complex cardiac anatomy—including aortic arch anomalies and pulmonary artery hypoplasia—distinguishing syndromic from non-syndromic TOF [35,36,37].

TOF also occurs in approximately 5–10% of individuals with Down syndrome, typically showing less severe right ventricular outflow tract obstruction but a higher prevalence of endocardial cushion and atrioventricular septal defects, reflecting perturbed mesodermal and endocardial signaling during cardiac septation [36,37].

Mutations in *JAG1* and, less commonly, *NOTCH2*, disrupt the Notch signaling pathway, leading to Alagille syndrome, where TOF is the most frequent congenital cardiac lesion. Patients may exhibit cholestasis, butterfly vertebrae, ocular anomalies, and characteristic facies, and diffuse pulmonary artery hypoplasia often complicates surgical repair [37].

Other syndromic contexts include CHARGE syndrome (*CHD7* mutations), Kabuki syndrome (*KMT2D* mutations), and Noonan spectrum disorders (*PTPN11*, *KRAS*, *SOS1*), all of which involve defects in neural crest migration, chromatin remodeling, and transcriptional regulation, highlighting the diverse molecular mechanisms underlying syndromic TOF [34,35,37].

### 5.3. Gene-Specific Anatomical Associations

Distinct genotypes have been linked to specific anatomic and extracardiac manifestations.

Variants in *FLT4* (VEGFR3) and *KDR* (VEGFR2), both key mediators of vascular and lymphatic signaling, are enriched in TOF cases with pulmonary valve hypoplasia, hypoplastic pulmonary arteries, and reduced pulmonary valve annulus dimensions, supporting their role in outflow tract and vascular remodeling [5,38]. *NOTCH1* mutations—among the most frequent monogenic causes of non-syndromic TOF—are associated with aortic root dilation and conotruncal malalignment, suggesting disruption of neural crest-derived cell signaling [4,33]. In contrast, *GATA4* and *HAND2* variants tend to produce milder anatomic forms with less severe RVOT obstruction [4,39]. *GATA6* mutations have been reported in TOF patients with pancreatic agenesis, diaphragmatic defects, or biliary malformations, indicating its role in both cardiac and endodermal development [4].

These findings underscore how perturbations in transcriptional and signaling pathways affect not only cardiac morphogenesis but also extracardiac organogenesis.

Importantly, despite these gene-specific tendencies, the current literature does not support the attribution of specific genes to distinct Fallot subtypes such as Trilogy or Pentalogy of Fallot. To date, no studies have defined a unique genetic basis for Trilogy of Fallot as an independent entity, highlighting a significant gap in knowledge and reinforcing the interpretation of Fallot-type malformations as a continuous and overlapping phenotypic spectrum rather than as genetically discrete categories.

## 6. Clinical Implications

### 6.1. Integrating Genetics into Clinical Follow-Up

The identification of a pathogenic variant can significantly influence clinical surveillance and management. For instance, carriers of *NOTCH1* or *FLT4* mutations may benefit from periodic imaging to monitor for aortic dilation or right ventricular outflow tract (RVOT) obstruction, while carriers of *JAG1* mutations require multisystemic follow-up due to hepatic and vascular involvement [10,38,39,40]. Similarly, in syndromic TOF, such as 22q11.2 deletion (DiGeorge), Kabuki syndrome, or 1p36 microdeletion, genetic diagnosis informs surveillance for multisystemic comorbidities—including endocrine, hematologic, neurodevelopmental, and immunologic complications—which may emerge throughout the patient’s life. Integration of genomic data into family counseling promotes a personalized approach to care, aligning with the growing emphasis on precision medicine in congenital heart disease [3,20,41]. Establishing a molecular diagnosis thus has direct implications not only for patient management, but also for anticipatory guidance and risk assessment in family members, whereas confirming isolated TOF provides reassurance regarding limited extracardiac involvement and lower recurrence risk.

### 6.2. Genetic Testing Strategy

The genetic evaluation of Tetralogy of Fallot (TOF) and other congenital heart diseases (CHDs) follows a structured, multidisciplinary workflow combining clinical and molecular approaches. According to the ESC 2020 Guidelines for Adult Congenital Heart Disease, the JCS/JCC/JSPCCS 2024 Guidelines on Genetic Testing and Counselling in Cardiovascular Disease, and the EHRA/HRS Expert Consensus Statement on Genetic Testing (2022), the process begins with a detailed personal and family history assessment, followed by pre-test genetic counseling as an essential step before any laboratory testing [41,42,43,44].

If syndromic or extracardiac features are present—such as dysmorphism, developmental delay, or known chromosomal syndromes—first-line testing should include a chromosomal microarray (CMA) ± fluorescence in situ hybridization (FISH), targeting pathogenic copy number variants such as the 22q11.2 deletion, which is well-established in TOF and conotruncal defects [36,42,44].

For isolated or non-syndromic TOF, the yield of CMA is lower, and a targeted gene panel or whole-exome/whole-genome sequencing (WES/WGS) is recommended when a monogenic etiology is suspected [4,5,6,14,38,40]. Recent genomic studies have identified major contributors including FLT4, NOTCH1, VEGF-related signaling genes, and MST1R, supporting a model of polygenic and pathway-level disruption in cardiac morphogenesis [4,5,6,12,13,14,38,39]. Epigenetic regulation (e.g., NOTCH4 methylation and Tbx1–Mef2c histone deacetylation) further modulates phenotypic expression, bridging the gap between genetic and developmental mechanisms [20,21,26].

**Figure 3:**
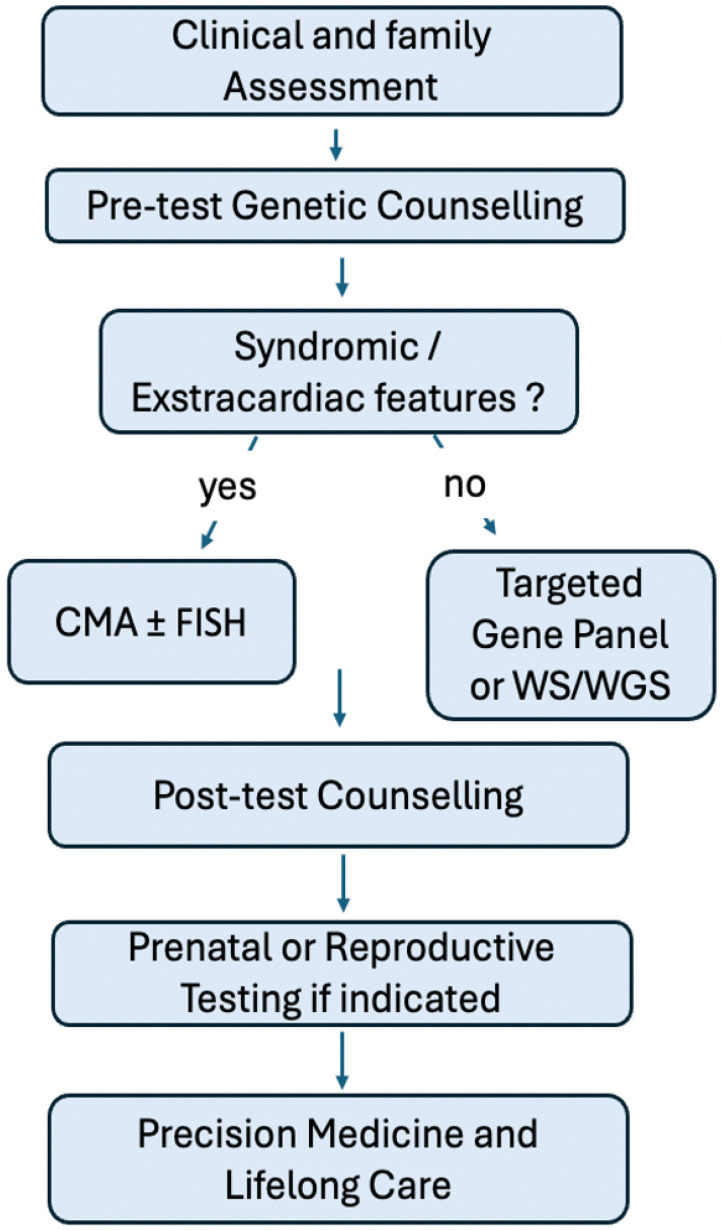
Genetic Testing Strategy.

Multidisciplinary involvement and post-test counseling are crucial to interpret results, assess familial recurrence risk, and guide cascade testing or surveillance in relatives [42,43,44]. In prenatal and reproductive contexts, particularly in families with prior affected offspring or identified variants, guidelines recommend offering CMA ± exome sequencing and discussing prenatal diagnostic or pre-implantation genetic testing (PGT) options [42,43,44] (Figure 3). The AHA/ACC 2018, ERA, and EHRA/HRS consensus similarly emphasize that identifying a molecular diagnosis can influence prognosis, follow-up strategy, and management, integrating precision medicine into lifelong CHD care [43,44].

Overall, these harmonized international recommendations advocate a tiered, phenotype-driven approach—beginning with counseling and chromosomal analysis in syndromic cases, advancing to exome/genome sequencing in isolated or familial disease, and extending to prenatal or preconception testing when indicated—to optimize patient outcomes through genetically informed cardiovascular care [3,42,43,44].

### 6.3. Family Counseling and Genetic Risk in TOF

Family counseling is a critical component of the clinical management of Tetralogy of Fallot (TOF), providing affected families with essential information on recurrence risks, inheritance patterns, and implications for future pregnancies. Although the majority of TOF cases are sporadic and multifactorial, advances in genomic medicine have revealed that up to 20–30% have an identifiable genetic or chromosomal cause, emphasizing the need for structured genetic counseling and family-based risk assessment [4,5,40].

The recurrence risk of TOF in siblings depends largely on whether a pathogenic variant or chromosomal abnormality is identified. Sporadic, non-syndromic TOF without a known genetic defect carries a low recurrence risk (≈2–5%), reflecting multifactorial inheritance mechanisms [37]. Autosomal dominant mutations, such as those in *NOTCH1*, *FLT4*, *NKX2-5*, or *GATA6*, may result in a 50% recurrence risk, though penetrance is variable and expressivity ranges from mild septal defects to classic TOF [4,5,40]. When TOF occurs as part of a chromosomal syndrome (e.g., 22q11.2 deletion, Alagille, or Down syndrome), the recurrence risk is typically <1%, unless a parent is a carrier of the structural rearrangement [36,40].

These estimates should always be interpreted within the context of family history, clinical features, and genetic test results, ideally supported by comprehensive pre- and post-test counseling.

### 6.4. Psychosocial and Ethical Considerations

Family counseling also addresses the psychological and ethical aspects of genetic testing, particularly regarding variants of uncertain significance (VUS), incomplete penetrance, and the implications for unaffected family members. Studies have shown that clear, multidisciplinary communication—combining cardiology, genetics, and psychology—improves family understanding and reduces anxiety related to reproductive decisions [41].

Moreover, cascade testing of at-risk relatives enables early detection of subclinical cardiac lesions and contributes to comprehensive family-based care [44].

## 7. Future Directions

Tetralogy of Fallot (TOF) arises from interactions between genetic mutations and epigenetic dysregulation, with epigenetic modifications influencing variable expressivity and penetrance [3,4,5,20]. Emerging technologies—single-cell transcriptomics, multi-omics, and iPSC/3D cardiac organoids—are clarifying how variants in *NOTCH*, *VEGF*, and *TBX1* pathways disrupt cardiac development and allow functional assessment of pathogenicity [4,5,6,12,13,14,38,39]. Integration of polygenic risk scores, epigenetic mapping, and machine-learning approaches may improve individualized risk prediction and patient management [12,13,20,21,26]. Future multicenter studies combining deep phenotyping and functional validation will advance predictive, preventive, and personalized cardiogenomics in TOF [42,43,44].

## 8. Conclusions

Tetralogy of Fallot (TOF) arises from a complex interplay of monogenic, oligogenic, and polygenic variants, along with epigenetic and environmental factors, reflecting the molecular heterogeneity of the disease. Multiple developmental pathways—including *NOTCH*, *VEGF/FLT4*, Wnt/Disheveled signaling, and neural crest interactions—contribute to the cardiac phenotype. Recent studies have also identified emerging candidate genes such as *PUF60*, *DVL3*, and *GPR91*, which may influence cardiac morphogenesis, neural crest development, and neurological vulnerability, expanding the phenotypic spectrum of TOF.

Comprehensive molecular diagnostics, including chromosomal microarray, targeted gene panels, and exome/genome sequencing, are essential for identifying pathogenic variants and for enabling personalized risk assessment, prognosis, and family counseling. Integrating genomic and bioinformatic analyses supports a precision medicine approach, improving understanding of disease mechanisms and informing tailored management strategies to optimize outcomes in TOF patients.

## Data Availability

No new data were created or analyzed in this study. Data sharing is not applicable to this article.
